# Prevention and Restoration of Hearing Loss Associated with the Use of Cisplatin

**DOI:** 10.1155/2014/925485

**Published:** 2014-07-22

**Authors:** Felician Chirtes, Silviu Albu

**Affiliations:** ^1^Otolaryngology Department, Military Hospital Cluj-Napoca, 22 General Traian Mosoiu Street, 3400 Cluj-Napoca, Romania; ^2^II-nd Department of Otolaryngology, Iuliu Hatieganu University of Medicine and Pharmacy Cluj-Napoca, Strada Republicii 18, 400015 Cluj-Napoca, Romania

## Abstract

*Background*. Cisplatin is a well known platinum-based chemotherapeutic agent used for the treatment of various malignant tumours. A frequent side effect of cisplatin therapy is ototoxicity. Unfortunately, currently there are no available treatments. *Material and Methods*. Experimental, clinical studies and reviews published between 2004 and 2014 in the English medical literature concerning ototoxicity were selected using Medline, PubMed, and Google Scholar databases. Inclusion criteria were cisplatin-induced ototoxicity and therapy aimed at preventing or curing this disorder. Molecular mechanisms and clinical, audiological, and histological markers of cisplatin-induced ototoxicity are described. Moreover, experimental and clinical strategies for prevention or treatment of hearing loss were also reviewed. *Results and Discussion*. Experimental studies demonstrate a wide range of otoprotective molecules and strategies efficient against cisplatin-induced hearing loss. However, only dexamethasone proved a slight otoprotective effect in a clinical study. *Conclusion*. Further research must be completed to bring future therapeutic options into clinical setting.

## 1. Introduction 

Cisplatin (cis-diamminedichloroplatinum II) is a well-known chemotherapeutic alkylating agent very effective in the treatment of various malignant tumors, especially squamous cell cancers of the head and neck regions, in both the pediatric and adult age groups [1]. Though synthesized already since 1845 by Peyrone owing to him its name of Peyrone's salt, only in the late 1960s was it used clinically in oncologic therapy for head and neck, lung, bladder, cervical, ovarian, testicular, and gastrointestinal cancers, as well as malignant gliomas and metastatic cancers such as melanoma, mesothelioma, and those of the prostate and breast [2]. One of its major side effects is irreversible neurosensorial hearing loss which affects ears symmetrically: high frequencies in the first place followed by low, speech range frequencies in a dose related and cumulative fashion. Cisplatin-induced hearing loss is favoured either by preexisting afflictions like hypoalbuminemia, anaemia, renal failure, and noise-induced hearing loss or by risk factors like therapy with loop diuretics, aminoglycoside antibiotics, radiotherapy fields which includes the inner ear, extreme ages (very young or very old), duration and dose schedule of cisplatin infusion, and genetic factors. Between 11% and 97% of patients treated with cisplatin develop hearing loss, with an average incidence of 62% [3]. Cisplatin-induced tinnitus is not an infrequent occurrence either. Ototoxicity may occur within hours to days after cisplatin administration. As subsequent multiple cisplatin regimens for the control of cancer may be necessary, side effects should be prevented or treated without reducing the efficacy of the antitumor mechanisms.

## 2. Material and Methods

A review of the literature from 2004 to 2014 was performed, using the Medline, PubMed, and Google Scholar databases. The search terms included cisplatin-induced hearing loss, protective therapy for inner ear diseases, intratympanic therapy, systemic therapy, and gene therapy. Only original experimental and clinical research papers were included. A total of 43 relevant papers were selected for the present review.

## 3. Results

### 3.1. Mechanisms of Cisplatin Ototoxicity

Cisplatin inflicts injuries mainly to outer hair cells, progressing from the third to the first row and to some extent to inner hair cells of the organ of Corti in the basal turn of the cochlea followed by alterations in sensorial cells situated in the apex. Cisplatin also targets supporting cells, marginal cells of the stria vascularis, and the spiral ligament [4]. The vestibular organs are not spared, nor are spiral ganglion cells in experimental conditions [5]. The ensuing hearing loss may be very disabling to patients whose communication is already impaired due to cancers of the head and neck. Hearing loss is also a common cause for depression and reduction of the quality of life [6].

Molecular mechanisms of cisplatin-induced hearing loss involve the creation of reactive oxygen species and depletion of antioxidant glutathione and its regenerating enzymes as well as increased rate of lipid peroxidation, oxidative modifications of proteins, nucleic acids damage by caspase system activation [3], and S-nitrosylation of cochlear proteins and resulting apoptosis of inner ear cells [7].

Oxygen free radicals in the cochlea are produced principally in the wake of nicotinamide adenine dinucleotide phosphate oxidase 3 isoform (NOX3) activation. NOX3 is known to be upregulated by cisplatin. Cochlear tissues fight the oxidative stress by means of antioxidant defence systems including glutathione, glutathione reductase, superoxide dismutase, and catalase [1]. Cisplatin-induced disturbance of potassium uptake and secretion in the stria vascularis has also been suggested, leading to impairment of the function of outer and inner hair cells in the organ of Corti, with alteration of the endocochlear potential and subsequent hearing loss [1]. A summary of mechanisms of cisplatin associated ototoxicity is displayed in Table 1.

The extent of ototoxicity due to cisplatin administration can be assessed clinically through measurements of hearing loss by means of pretreatment and follow-up serial audiologic tests and experimentally through histological examinations [8]. The former include pure tone (normal frequency and extended high-frequency range), speech and impedance audiometry, auditory brainstem responses, and Distortion Product Otoacoustic Emissions (DPOAEs) testing. DPOAEs reflect early injuries to outer hair cells in the organ of Corti thereby allowing monitoring and early detection of cisplatin ototoxicity during cancer treatment [9, 10]. Cisplatin early ototoxic effects cause hearing impairment in the high frequencies at 6 kHz, 8 kHz, and above as measured by conventional or extended high-frequency pure tone audiometry [11]. Multiple doses of cisplatin worsen hearing, ultimately affecting the speech frequency range (500–4000 Hz). Since DPOAEs measurement is based on the integrity of outer hair cells which is affected by cisplatin therapy before elevation of auditory threshold as measured by pure tone audiometry, DPOAE testing is more sensitive than the latter for the detection of cisplatin-induced ototoxicity. Moreover, the results provided by DPOAEs, as an objective testing, are not influenced by the ability of the deteriorating cancerous patient to respond to the sound stimulus [9]. In small children who undergo cisplatin administration for cancer treatment and who cannot cooperate for pure-tone testing because of their younger age and poor cognitive ability, either sound field behavioural testing or DPOAEs can be performed [12].

In experimental animals, hearing loss after cisplatin treatment can be assessed by audiological study of the auditory brainstem responses where threshold measurement defines the lowest intensity of sound stimulus that evokes a clear, visually detectable, reproducible waveform [4].

Histologic examinations of inner ear after cisplatin administration reveal destruction primarily of outer hair cells and to some extent inner hair cells and associated nerves, degeneration of the vestibular organs, stria vascularis edema, and detachment of the myelin sheath of the spiral ganglion cells [5]. Light microscopy of cochlear samples obtained from animals receiving cisplatin shows loss of hair cells with collapse of the tunnel of Corti and Nuel's space. In cisplatin treated animals' cochlea the scanning electron microscopy detects damage and loss of stereocilia of the hair cells as well as rupture of the cuticular plate [13]. The damage is especially noticed in the high frequency region of the cochlea (i.e., the basal turn) probably due to a base to apex gradient of the cisplatin ototoxicity [14]. Tumour necrosis factor-alpha (TNF-*α*) and other inflammatory cytokines (IL-8, IL-6) were also detected by immunostaining in the outer hair cells, stria vascularis, spiral ligament, and spiral ganglion neurons in cisplatin exposed cochleae [4].

Cisplatin pharmacokinetics in the inner ear after intravenous injection is influenced by its strong binding to the plasma proteins rendering a large part of it nonactive and by the barrier systems in the cochlea, the blood-perilymph barrier, separating blood from perilymph, and the intrastrial fluid-blood barrier, separating blood from endolymph [15]. The amount of free chemotherapeutic agent reaching targets in the cochlea is responsible for the ototoxic effect and consequent hearing loss. High frequency audiometric thresholds are initially affected. When doses in excess of 100 mg/m^2^ are used the hearing impairment may progress from high frequencies to involve the middle frequencies. Reducing the total amount of cisplatin by limitation of the total dose per cycle, dose intensity and the cumulative dose would diminish the antitumor effect which is not desirable. Various otoprotective compounds have been tested both in experimental animals and humans. If given systemically, they should be nontoxic, must attain efficient concentrations in the inner ear to protect labyrinthine tissues from cisplatin ototoxicity, and should not hamper the antitumor effect of cisplatin. Higher concentrations of otoprotective molecules can be achieved by intratympanic administration. This latter route provides direct access of the protective agents to inner ear structures while avoiding systemic side effects and interference with the antineoplastic activity of cisplatin [16].

### 3.2. Experimental Studies

Several otoprotective molecules and strategies against cisplatin-induced ototoxicity have been tested in experimental animals. Some, like diethyldithiocarbamate, exerted important side effects in humans [16]. A summary of preventative and restorative treatment options is presented in Table 2.

Hyperbaric oxygen therapy consists of intermittent inhalations of 100% oxygen at a pressure higher than 1 atm and is used as adjuvant therapy in pathological processes like soft tissue infections, radiation injury, gas gangrene, and decompressive disease. Its main effect is tissue hyperoxygenation through plasma dissolved oxygen and was successfully tested in guinea pigs as a protective agent against cisplatin ototoxicity. Efficacy of otoprotection by hyperbaric oxygen therapy after cisplatin administration was evaluated by otoacoustic emissions following hyperbaric treatment and by scanning electron microscopy examination. The study confirmed that cisplatin induces dose-dependent cochlear alterations consisting of cellular lesions and significant hair damage in outer hair cells. Analysis of anatomical changes in cochlear outer hair cells indicated signs of otoprotection against cisplatin in animals treated with hyperbaric oxygen therapy although in functional studies distortion product acoustic emission were absent reflecting a certain degree of hearing loss, most probably reversible and related to experimental artefacts. The study concluded that hyperbaric oxygen therapy has otoprotective effects against cisplatin-induced ototoxicity. However, further studies are necessary to test other effects of high pressure oxygen on the cochlea [17].

Another study investigated the effect of epigallocatechin gallate (EGCG) on the transcription factor STAT1, an important mediator of cell death. STAT1 phosphorylation is involved in both hair cells and support cells transformation after experimental exposure of mouse utricule to cisplatin. EGCG proved its efficiency as an otoprotective agent against cisplatin ototoxicity due to its inhibition of STAT1. The hypothesis was further supported by the failure of EGCG to provide protection against cisplatin in STAT1-deficient mice [18].

Former studies showed that lactate injected intratympanically in guinea pigs treated with ototoxic levels of cisplatin allowed near total preservation of otoacoustic emissions [19]. Since lactate is a part of Ringer solution, safely used in human subjects, and has the smallest molecular weight among other antioxidants which facilitates transport across the round window membrane, a further study was conducted to prove its otoprotective effect against cisplatin ototoxicity when injected intratympanically, before intraperitoneal cisplatin administration. The molecular protective mechanism is based on the enzyme lactate dehydrogenase located in the mitochondria of outer hair cells. The conversion of lactate to pyruvate in the presence of the enzyme leads to the formation of nicotinamide adenine dinucleotide (NADH) which is a natural antioxidant that may be involved in reducing toxic effects of oxygen reactive species resulted at cellular level following cisplatin therapy. Electron microscopy examinations of guinea pig cisplatin-insulted inner ears pretreated with lactate showed partial preservation of outer hair cells stereocilia, more significant at midfrequencies (2000–4000 HZ) but not statistically significant at higher frequencies. The study used auditory brainstem responses recordings which is a more sensible method than otoacoustic emissions testing, a fact that explains the differences in otoprotective effect reported by previous studies. The same study investigated the otoprotective effect of intratympanic N-acetylcysteine injections as well as its systemic diffusion following the administration. The results showed that high concentration of intratympanic N-acetylcysteine is not reliable for otoprotection against cisplatin ototoxicity since it caused more middle and inner ear damage than cisplatin alone. Yet, N-acetylcysteine did not diffuse systemically when applied to the middle ear. This was confirmed by high-performance liquid chromatography testing of blood samples taken from the venous system of the experimental animals after intratympanic injections of N-acetylcysteine. This latter outcome proves that the intratympanic route of administration would be safe and prevent inactivation of antitumor effect of cisplatin by binding between the thiol moiety of N-acetylcysteine and the platinum-containing molecule of the chemotherapeutic drug [20].

Most of the existing studies focus on exogenous administration of antioxidants. Pharmacological activation of intrinsic defence mechanisms against oxidative stress in the inner ear caused by cisplatin therapy also proved helpful as showed by an experimental animal study using systemic administration of thiamine pyrophosphate (TPP). Thiamine pyrophosphate functions as coenzyme for peroxisomes being a crucial factor for energy metabolism, antioxidation, and myelinisation of nerve cells. Its intraperitoneal injection increased the level of natural antioxidants like glutathione and antioxidant enzymes (superoxide dismutase, glutathione peroxidase and glutathione reductase) and reduced the content of malonildialdehyde, an indicator of lipid peroxidation following increased levels of oxygen reactive species resulting from cisplatin toxicity. The histologic evaluation of cochleae harvested from TPP treated animals showed preservation of the morphology of the organ of Corti and outer hair cells and no destruction of spiral ganglion cells and stria vascularis following cisplatin therapy [5].

The wide range of therapeutic molecules studied for their otoprotective effect against cisplatin-induced ototoxicity also includes sertraline, an antidepressant with neuroprotective effects in rats [6]. The selective serotonin reuptake inhibitor also has antioxidant effects, stimulates neurogenesis, and increases antiapoptotic protein levels [21]. It has been documented by distorsion product otoacoustic emissions recordings that oral administration of sertraline in cisplatin-treated rats prevented hearing loss above 5000 Hz, in a statistically significant manner. Besides, sertraline would be beneficial to patients whose communication abilities are already deteriorated either by the cancer itself or by the treatment modalities and, therefore, feel depressed [6].

An experimental single dose model of cisplatin ototoxicity in guinea pigs showed the otoprotective effect of systemic histone deacetylase inhibitor sodium butyrate. Distortion product otoacoustic emissions testing were chosen to provide a sensitive assay of the functional state of outer hair cells after systemic cisplatin insult on the cochlea. The systemic administration of the otoprotective agent avoided the side effects of the more invasive tympanic local route of administration. Moreover, sodium butyrate did not interfere with the tumoricidal effect of cisplatin providing both protections from reactive oxygen species and a certain degree of antitumor activity according to former reports. Acetylation of different cell proteins, including histones, is responsible both for the protective effect against oxidative stress and for the cell division inhibition and subsequent anticancer activity. The experiment's weak point is that it only showed the effect of sodium butyrate in a single dose of cisplatin model whereas in clinical practice cisplatin is typically given repeatedly at a couple of weeks intervals for several months [22].

The unique isoform of NADPH oxidase, NOX3, found in the cochlea and its involvement in the generation of reactive oxygen species was at the base of an animal study showing the efficacy of short interfering RNA in preventing cisplatin ototoxicity by reducing the expression of NOX3 in outer hair cells, spiral ganglion cells, and stria vascularis in the rat. Auditory brainstem responses were used to certify reduced threshold shifts in cisplatin treated animals who received transtympanic NOX3 siRNA. Since cisplatin administration has been previously associated with upregulation of NOX3 in the inner ear, Nox3 is thought to be a major source of free radicals in the cochlea following cisplatin exposure. The resulting free radicals initiate the inflammatory process in the cochlea by activating signal transducer and activator of transcription-1 (STAT1), followed by activation of p53 and increase in inflammatory mediators like TNF-alpha and interleukin-1*β* [23]. A single transtympanic injection of siRNA attenuated cisplatin ototoxicity by suppressing inflammation in a dose-related manner. It hampered cisplatin-induced auditory brainstem responses threshold shift and higher doses allowed for complete morphological preservation of outer hair cells as proven by scanning electron microscopy examinations of the rat cochleae [24].

Among various strategies that have been devised in experimental settings to prevent cisplatin ototoxicity, minocycline, a tetracycline derivative, proved its partial efficacy in vivo and in vitro. The anti-inflammatory and neuroprotective properties of minocycline have been previously reported. The biochemical mechanisms involve caspase-1 and caspase-3 inhibition, which decreases the amount of interleukin-1 and prevents apoptosis. The protective effect of minocycline has been tested both on cisplatin treated cell cultures and in experimental animals which underwent cisplatin intraperitoneal therapy after systemic administration of the otoprotective agent. Cell viability assays showed that minocycline had a protective effect against cisplatin toxic action. Yet, minocycline failed to protect cells at higher concentrations of cisplatin. Recordings of auditory brainstem responses and evaluation of the scanning electron microscopy sections of inner ears harvested from minocycline plus cisplatin treated animals indicated a partial preservation of the function and morphology of the outer hair cells as compared to those from animals treated with cisplatin alone [25].

The effect of intraperitoneal administration of erdosteine on cisplatin-induced ototoxicity in a guinea pig model was also studied. Erdosteine is a thiol derivative with established antioxidant properties due to its active sulfhydryl groups following liver first-pass metabolism. Pre- and posttreatment auditory brainstem responses measurements were performed in living animals while outer hair cell counts were analyzed by scanning electron microscopy of the cochleae removed from euthanized animals. Although the study had limitations (i.e., minimal number of animals included, lack of enzymatic activity detection for the main antioxidant enzymatic systems of the inner ear), the results outlined the systemic administration of erdosteine as a promising therapeutic strategy for cisplatin-induced ototoxicity [26].

In a first murine model for cisplatin-induced ototoxicity, it was shown that intratympanic dexamethasone prevents hearing loss in a frequency related manner. Evoked brainstem responses audiometry indicated that 8 kHz and 16 kHz stimulus elicited responses in cisplatin plus dexamethasone treated mice while high frequency stimulus (32 kHz) perception was affected. Apparently, cisplatin had deleterious effects on outer and inner ear cells situated in the basal turn of the cochlea despite intratympanic administration of protective dexamethasone [27]. Further experimental studies supported the finding that cisplatin exerts its damaging effect in a base to apex gradient, lower frequencies being spared for long. Higher doses of dexamethasone also seem to be more protective than lower doses. Moreover, lower doses of cisplatin allow the naturally present antioxidants to annihilate the resulting reactive oxygen species, explaining the spontaneous hearing threshold recovery even in the absence of protective dexamethasone administration [28].

The intratympanic administration of dexamethasone avoids diminishing the tumoricidal activity of cisplatin. Downregulating apoptosis genes in tumour cells are responsible for this common side effect of systemic steroid therapy [29]. An experimental study conducted on cisplatin treated guinea pigs asserted the safety of intratympanic dexamethasone based on audiologic and histologic results. Auditory brainstem responses testing, optic microscopic and scanning electron microscopic examinations of cochleae showed no significant differences between intratympanic dexamethasone-treated animals and saline-treated controls. Dexamethasone administered intratympanically proved efficacious in protecting the labyrinth against cisplatin-induced ototoxicity as shown by reduced auditory brainstem responses threshold shifts and unaltered histological inner ear structures. The molecular mechanisms involve increased expression of Na/K channels and aquaporins in the endolymphatic sac and the tissues around the endolymphatic spaces. The study's results also suggest that giving the intratympanic dexamethasone one hour before the cisplatin administration provides the best protection (total protection) against the ototoxic insult by the alkylating agent compared to dexamethasone injections one day prior to cisplatin administration (partial protection) [14]. According to another study, in order for the dexamethasone to exert a protective effect against cisplatin ototoxicity, the timing of administration of the two drugs should be highly synchronized so that the peak concentration of dexamethasone in the perilymph should correlate with the peak concentration of the chemotherapeutic agent [3].

Otoprotection with dexamethasone against cisplatin-induced age-related hearing loss was investigated in a guinea pig model following observations that persons older than 65 years account for more than half of the newly diagnosed malignancies. Hearing loss due to the aging process shares the same cause (i.e., oxidative stress) with hearing loss due to ototoxic chemotherapeutic agents. A single dose of cisplatin was administered intraperitoneally in old mice preceded and followed by dexamethasone injected intratympanically to counteract the cisplatin toxic effect on inner ear hair cells. Pre- and posttreatment auditory brainstem responses were recorded to evaluate hair cell function. The results of the study pointed out that no synergistic action between age related hearing loss and cisplatin-induced hearing loss exists since threshold shifts were smaller in older animals than those in young mice. Another finding of the study was that the protective effect of dexamethasone against cisplatin-induced ototoxicity was a function of stimulus frequency in old mice. Susceptibility to otoprotective effect of dexamethasone was higher in mid to basal cochlear regions (at and above 24 kHz) in old mice, whereas in young mice, dexamethasone bestowed more protection in apical regions of the cochlea (at 16 kHz and below). Age-related changes of the mechanism of distribution of dexamethasone in scala tympani perilymph after round window membrane application in guinea pigs seem to account for the frequency dependent otoprotective effect [30].

Intratympanic dexamethasone failed to protect against cisplatin-induced ototoxicity in a multidose cisplatin ototoxicity mouse model. The study was prompted by typical clinical protocols of cancer treatments which require administration of multiple, smaller cisplatin doses, exerting their curative effect through cumulative dosing. Contrary to previous experimental studies, the mice received five doses of cisplatin throughout five days, mimicking the cumulative exposure seen in malignant tumours treatment. Intratympanic dexamethasone was administered on the same days as the intraperitoneal cisplatin. The results mirrored by auditory brainstem responses threshold measurements demonstrated continued change in hearing thresholds several weeks after cisplatin exposure and no protective effect of intratympanic dexamethasone against cisplatin ototoxicity [31].

An experimental study focused on systemic administration of steroid for protection against cisplatin-induced ototoxicity showed no otoprotection following several days' prophylaxis with a high dose dexamethasone treatment. Only a slight decrease of TNF-alpha expression in the cochlea was demonstrated by immunohistochemical staining of anatomical samples harvested from systemic cisplatin plus dexamethasone treated animals. Dexamethasone also seemed to protect stria vascularis from morphological alterations, probably owing this effect to higher concentrations of steroid in the lateral cochlear wall following increased cochlear flow and a naturally highly vascularised stria vascularis. Still, a functional otoprotective effect of systemic dexamethasone against cisplatin-induced hearing loss was not observed [4].

Among naturally occurring molecules, Rosmarinic acid, a water-soluble polyphenolic compound extracted from Dansam-Eum, was tested for its protective effect against cisplatin-induced ototoxicity in laboratory settings. The results of the study showed that Rosmarinic acid inhibited cisplatin-induced caspase-1 activation providing protection against stereocilia loss in the primary organ of Corti explants [32].

Another natural remedy, the Maytenus ilicifolia aqueous extract, was evaluated for its possible otoprotection in guinea pigs. Despite the well-known South America plant's antioxidant effects (due to the presence of flavonoids and alkaloids), functional tests did not demonstrate any protective action on the cisplatin exposed cochleae. Yet, the extract improved the clinical status and weight of guinea pigs and diminished mortality after cisplatin exposure [33].

Resveratrol, a polyphenol found in grape skin and seed, has antioxidant, neuroprotective, and dose dependent antiapoptotic properties [34]. Recent experimental research pointed out the preventive effect of resveratrol against cisplatin induced ototoxicity. An in vitro study on House Ear Institute-Organ of Corti 1 cell line showed that resveratrol in low doses prevented ototoxicity mainly influencing apoptotic gene expression but proved cytotoxic effect in high doses [34]. Two other studies showed conflicting results. Thus, high doses of oral resveratrol administered to mice seem to enhance cisplatin ototoxicity [35] whereas systemic administration of lower doses of resveratrol provided significant protection to the cochlea against cisplatin [36].

### 3.3. Clinical Studies

Intratympanic dexamethasone was clinically tested for its otoprotective effect in patients suffering from neoplastic diseases for which the treatment protocol included cisplatin. Intratympanic dexamethasone has already been tested clinically for the treatment of idiopathic sudden sensorineural hearing loss and Meniere disease [37]. Intratympanic administration of steroids avoids significant systemic side effects like hyperglycaemia, peptic ulcers, hypertension, osteoporosis, and psychosis. The intratympanic route also provides higher concentrations of drug in the inner ear fluids and prevents significant interference between dexamethasone, which is known to reduce efficacy of chemotherapeutic agents, and cisplatin. Patients enrolled in the study underwent unilateral intratympanic dexamethasone administration prior to every cisplatin treatment session, with the contralateral ear used as a control. Serial follow-up audiometry and distorsion product otoacoustic emissions testing were performed to check the functional state of both study and control ears. The statistically significant results showed that intratympanic dexamethasone is slightly protective against cisplatin-induced hearing loss at 6000 Hz and decreases the outer hair cells dysfunction in the frequency range of 4000 to 8000 Hz. The conclusion of the study is that intratympanic dexamethasone has minimal effect towards reducing cisplatin ototoxicity. Further studies using different concentrations of dexamethasone and a perfect timing of administration are necessary to investigate its role in preventing hearing loss after cisplatin therapy [3].

Transtympanic L-N-acetylcysteine was also clinically tested in head and neck cancer patients undergoing cisplatin therapy. Thiol compounds are known to either directly bind cisplatin or act as free radical scavengers. Based on that, their intratympanic administration was suggested to avoid the decrease of oncologic effectiveness of cisplatin and to reduce the oxidative stress caused by it. Intratympanic L-N-acetylcysteine was well tolerated by patients receiving multiple doses of cisplatin as part of their oncologic treatment. The relation between dose and otoprotection was not taken into account. Higher concentrations may have yielded better otoprotection. The study protocol required the L-NAC injection to be approximately 1 hour before systemic administration of cisplatin, for the sake of better timing. The outcome of the pure tone audiometry testing at 1 and 2 months after the last cycle of cisplatin showed that L-N-acetylcysteine was overall not significantly otoprotective. Still, hearing loss was reduced in two patients out of eleven who completed the study. The study protocol had several challenges like the difficulty in maintaining high enough concentrations of aqueous solution of L-N-acetylcysteine in the middle ear due to the technique of administration. Another study flaw was the different initial hearing thresholds due to preexisting hearing loss [38].

Few clinical studies tested systemic otoprotective molecules for preventing cisplatin-induced hearing loss. Amifostine, a phosphorylated aminothiol designed to protect against radiation damage, was known to counteract the toxic effect of different anticancer treatments without interfering with the tumoricidal effect. Although significant protection of amifostine against haematological toxicity after high dose carboplatin therapy in a child with medulloblastoma was reported [39], a clinical study considering systemic administration of amifostine failed to prove any otoprotective effect against cisplatin-induced hearing loss in a group of pediatric patients treated with cisplatin associated with other chemotherapeutic agents [40]. Clinical trials currently underway as documented by their registration in a public database (http://clinicaltrials.gov/ct2/results?term=cisplatin+ototoxicity/) are listed in Table 3.

### 3.4. Otopharmacogenetics

The well-isolated inner ear organ makes it prone to targeted genetic therapies. Viral or nonviral gene vectors can be delivered through a transtympanic route without the risk of dispersing them and reaching other tissues with subsequent undesirable genetic alteration. Long term effects after single administration, cellular selectivity, and replacement of genetically flawed nucleic acid sequences are the main benefits of gene therapy. Common viral vectors include herpes simplex virus, recombinant adenoassociated virus, recombinant adenovirus, and adenovirus, used to amplify the expression of targeted genes. Cells are infected with the vectors (transfection) which transfer genes whose expressed proteins influence important processes like growth, oxidative stress, and apoptosis. Chemical transfection can also be achieved with plasmid vectors. Short interfering RNA can be used to shut down target genes.

Among inner ear target genes dealt with by the gene therapy studies are ATOH1 (Math1), CAT (catalase), SOD1 (Cu/Zn superoxide dismutase), SOD2 (Mn superoxide dismutase), BDNF (brain-derived neurotrophic factor), HGF (hepatocyte growth factor), GJB2 (gap junction protein), Bcl-xL (B-cell lymphoma-extra large), FGF2 (basic fibroblast growth factor). The gene therapy modifies the synthesis of a wide range of proteins including neurotrophic factors (NTF3, GDNF), apoptosis mediators (XIAP, BCL2), oxidases (NADPH, NOX1, NOX3, NOX4), an antioxidant response regulator (Nfe2l2), a cytoprotective enzyme (HO-1), copper transporters (Ctr1), a nonselective cation channel (Trpv1), and protein Otospiralin (Otos).

Cisplatin-exposed tissues can benefit from genetically induced upregulation of neurotrophic factors, inhibition of apoptosis, and generation of endogenous antioxidant enzymes.

Experimental animal studies and in vitro experiments show the efficacy of gene therapy for cisplatin-induced ototoxicity. Clinical applications require further studies regarding safety, immunogenicity, and consequences of genetic manipulation [41].

Another strategy to avoid cisplatin-induced hearing loss would be the pretreatment genotyping to find out patients at risk for the ototoxic effect of cisplatin [42]. Genetic variants (polymorphism) of different protein systems (Thiopurine S-methyltransferase, Catechol-O-methyl transferase, Glutathione-S-transferase with its subclasses M1/T1/P1, Magalia) can stand for the interindividual variability in cisplatin ototoxicity [43].

## 4. Discussion

Forty-three publications were reviewed concerning prevention or treatment of cisplatin induced ototoxicity. Publications were devised in either experimental or clinical studies. Experimental studies sustained the efficiency of hyperbaric oxygen therapy, epigallocatechin therapy, and intratympanic lactate. The latter two therapies provide exogenous antioxidants while pharmacologic activation of endogenous antioxidants by means of intratympanic thiamine pyrophosphate was consistent with higher levels of natural antioxidants. Oral sertraline, besides its otoprotective effect against cisplatin induced ototoxicity, also has therapeutic value concerning the depression occurring frequently in oncologic patients. Sodium butirate proved its efficiency against cisplatin induced hearing loss in a monodose cisplatin model. Yet, in clinical practice the patient receives multiple doses of cisplatin. The production of endogenous radicals of oxygen species was reduced after intratympanic administration of short interfering RNA which reduces the expression of NOX3 in the cochlea. Minocycline appeared to be efficient only at low doses of cisplatin while systemic erdosteine showed promising results. Dexamethasone in experimental studies combined efficiency against cisplatin induced ototoxicity with preservation of the tumoricidal activity of cisplatin. When older mice were treated, the dexamethasone was more otoprotective at higher frequencies compared to experiments including younger subjects. Rosmarinic acid also proved to be otoprotective while the Maytenus ilicifolia aqueous extract was not. Resveratrol had contradictory effects, systemic low doses, and in vitro administration preventing ototoxicity, whereas high oral doses seem to enhance cisplatin ototoxicity. There also were experimental studies which showed inefficiency of intratympanic N-acetylcysteine and intratympanic and systemic dexamethasone. N-Acetylcysteine also had a damaging effect on middle and inner ear structures.

Clinical studies proved a minor otoprotective effect of intratympanic dexamethasone and no effect of systemic amifostine and intratympanic L-N-acetylcysteine. New perspectives are brought about by genetic therapy using viral vectors and genotyping to anticipate interindividual variability in cisplatin ototoxicity.

## 5. Conclusion

Hearing loss prevention and treatment during cisplatin therapy for cancer needs further research to find new strategies and optimize old ones. The intratympanic route of administration along with the gene therapy appears to be the most attractive objective for further experimental and clinical studies.

## Figures and Tables

**Table 1:** Mechanisms of ototoxicity.

Cellular mechanisms of ototoxicity	Molecular mechanisms of ototoxicity
(i) Damage to outer hair cells (ii) Damage to supporting cells (iii) Damage to marginal cells of stria vascularis (iv) Damage to the spiral ligament (v) Damage to spiral ganglion cells	(i) Creation of reactive oxygen species (ii) Depletion of antioxidant glutathione and its regenerating enzymes (iii) Increased rate of lipid peroxidation (iv) Oxidative modifications of proteins (v) Nucleic acids damage by caspase system activation (vi) S-Nitrosylation of cochlear proteins

**Table 2:** Treatment of cisplatin ototoxicity.

Preventive treatment for cisplatin ototoxicity	Restorative treatment for cisplatin ototoxicity
Treatment of hypoalbuminemia, anemia, renal failure Intratympanic dexamethasone Transtympanic L-N-acetylcysteine Resveratrol	Thiol compounds Sertraline Hyperbaric oxygen therapy

**Table 3:** Clinical trials currently underway (http://clinicaltrials.gov/ct2/results?term=cisplatin+ototoxicity).

Trial title	Trial status
Protection from cisplatin ototoxicity by lactated Ringers	Completed
Alpha-Lipoic acid in preventing hearing loss in cancer patients undergoing treatment with cisplatin	Completed
The protective effect of Ginkgo Biloba extract on cisplatin-induced ototoxicity in humans	Completed
Preventing nephrotoxicity and ototoxicity from osteosarcoma therapy	Recruiting
Sodium thiosulfate in preventing hearing loss in young patients receiving cisplatin for newly diagnosed germ cell tumor, hepatoblastoma, medulloblastoma, neuroblastoma, osteosarcoma, or other malignancies	Active, not recruiting
SPI-1005 for prevention and treatment of chemotherapy induced hearing loss	Not yet recruiting
